# Evaluating the effect of voxel size on the accuracy of 3D volumetric analysis measurements of brain tumors

**DOI:** 10.3389/fradi.2025.1618261

**Published:** 2025-08-06

**Authors:** Rithvik S. Ghankot, Manwi Singh, Shelby T. Desroches, Noemi Jester, Amit Mahajan, Samantha Lorr, Frank D. Buono, Daniel H. Wiznia, Michele H. Johnson, Steven M. Tommasini

**Affiliations:** ^1^ School of Engineering and Applied Science, Yale University, New Haven, CT, United States; ^2^ School of Medicine, University of Sheffield, Sheffield, United Kingdom; ^3^ Department of Radiology and Biomedical Imaging, Yale School of Medicine, New Haven, CT, United States; ^4^ Department of Psychiatry, Yale School of Medicine, New Haven, CT, United States; ^5^ Department of Orthopedics, Yale School of Medicine, New Haven, CT, United States

**Keywords:** neurofibromatosis type 2 related schwannomatosis, voxel size, dice score, Hausdorff distance, AI, segmentation

## Abstract

**Introduction:**

Neurofibromatosis type 2 related Schwannomatosis (NF2-SWN) is a genetic disorder characterized by the growth of vestibular schwannomas (VS), which often leads to progressive hearing loss and vestibular dysfunction. Accurate volumetric assessment of VS tumors is crucial for effective monitoring and treatment planning. Since tumor growth dynamics are often subtle, the resolution of MRI scans plays a critical role in detecting small volumetric changes that inform clinical decisions. This study evaluates the impact of MRI voxel resolution on the accuracy of manual and AI-driven volumetric segmentation of VS in NF2-SWN patients.

**Methods:**

Ten patients with NF2-SWN, totaling 17 tumors, underwent high-resolution MRI scans with varying voxel sizes on different MRI machines at Yale New Haven Hospital. Tumors were segmented using both manual and AI-based methods, and the effect of voxel size on segmentation precision was quantified through volume measurements, Dice similarity coefficients, and Hausdorff distances.

**Results:**

Results indicate that larger voxel sizes (1.2 × 0.9 × 4.0 mm) significantly reduced segmentation accuracy when compared to smaller voxel sizes (0.5 × 0.5 × 0.8 mm). In addition, AI-based segmentation outperformed manual methods, particularly at larger voxel sizes.

**Discussion:**

These findings highlight the importance of optimizing voxel resolution for accurate tumor monitoring and suggest that AI-driven segmentation may improve consistency and precision in NF2-SWN tumor surveillance.

## 1 Introduction

Neurofibromatosis type 2 related Schwannomatosis (NF2-SWN) is an autosomal dominant disorder caused by mutations in the NF2-SWN tumor-suppressor gene on chromosome 22. This condition is characterized by the development of multiple nervous system tumors, particularly meningiomas and vestibular schwannomas (VS), often leading to progressive hearing loss and vestibular dysfunction (1, 2). NF2-SWN management primarily relies on serial annual surveillance imaging to track tumor progression and evaluate risks to adjacent critical structures, given the limited effectiveness of pharmacologic, radiation, and surgical treatments. Accurate volumetric assessment is essential for early intervention and treatment planning, as subtle changes in tumor size can have significant clinical implications.

Traditionally, VS tumor progression is evaluated using volumetric analysis based on ellipsoid modeling, as recommended by the Response Evaluation in Neurofibromatosis and Schwannomatosis (REiNS) consortium (3). This approach estimates tumor volume using the length and width of the largest cross-section, as determined by a neuroradiologist. However, studies exploring volumetric analysis of VS tumors have found that ellipsoid approximations overestimate volume (Yamada et al., 2000). As VS tumors grow, their cross-sectional morphology often deviates from a simple ellipsoid, particularly due to the development of extra-canalicular extensions into the cerebellopontine angle. This results in a characteristic ‘ice cream cone’ shape, which may contribute to volume overestimation when using ellipsoid-based models (4).

A more precise alternative is 3D volumetric analysis, which has been applied to NF2-SWN-associated VS (5). This technique reconstructs tumor volume by segmenting its area across all MRI slices, generating a highly accurate 3D representation of the tumor's shape and spatial relationships with surrounding structures. This method provides greater precision in assessing tumor burden and may help predict the onset of clinical symptoms based on anatomical involvement. However, manual 3D segmentation remains time-intensive and prone to interobserver variability, which can impact the reliability of longitudinal tumor assessments. Recent advancements in artificial intelligence (AI)-driven auto-segmentation tools have further improved efficiency and accuracy (6, 7). By reducing interobserver variability and segmentation time, AI-based volumetric analysis offers a more consistent and reproducible approach to tracking tumor growth over time.

While AI-driven segmentation enhances consistency, the accuracy of any 3D tumor model is ultimately constrained by the resolution of the source MRI data, which is dictated by voxel size. Voxel volume is influenced by the field of view, matrix size, and slice thickness (8):

$$\mathrm{Voxel}\;\mathrm{Volume}\;=\frac{\mathrm{Field}\;\mathrm{of}\;\mathrm{View}}{\mathrm{Matrix}\;\mathrm{Size}}\;\times \;\mathrm{Slice}\;\mathrm{Thickness}$$

Here, the field of view represents the scanned area, the matrix size defines the number of voxels per field of view, and the slice thickness determines the depth of each cross-section. Higher matrix sizes yield smaller voxel dimensions and improved resolution, while larger slice thicknesses may reduce accuracy in volume estimation. Understanding these imaging parameters is crucial for optimizing tumor segmentation techniques, whether performed manually or by AI.

This study evaluates the impact of MRI voxel size on the accuracy of manual and AI-driven volumetric segmentation of vestibular schwannomas in NF2-SWN. Given that tumor progression influences clinical decision-making—including the timing of surgery or radiation therapy—precise volumetric assessment is essential. However, current methods face limitations: ellipsoid approximations introduce overestimation errors, while manual 3D segmentation remains time-consuming and variable. AI-based tools offer improved efficiency, but their accuracy depends on underlying MRI resolution, which is dictated by voxel dimensions (field of view, matrix size, and slice thickness). By quantifying how voxel size affects segmentation precision, this work aims to establish empirically supported imaging parameters that balance accuracy with clinical feasibility. Optimizing volumetric analysis may enhance early detection of tumor progression, support more informed treatment decisions, and contribute to better long-term outcomes for NF2-SWN patients.

## 2 Methods

### 2.1 Sampling

Ten randomly selected NF2-SWN patient MRIs from Yale New Haven Health, all of whom had undergone annual brain MRIs for vestibular schwannoma surveillance, were included in this study. The cohort had a mean age of 49.5 years (range: 22–65 years) and consisted of seven females and three males; two patients identified as Hispanic. The NF2-SWN mutation was familial in two cases and mosaic in eight. A total of 17 tumors (seven bilateral and three unilateral VS) were analyzed. Five patients had no prior surgical intervention, while the remaining five had undergone a single surgery each, including two gamma knife radiosurgeries and three open resections. This retrospective study was approved by the Yale University Institutional Review Board. Informed consent was waived due to the use of de-identified data. A formal power analysis was not conducted due to the exploratory nature of this pilot study. Future work will involve larger, statistically powered cohorts to enable robust subgroup analysis.

Inclusion criteria for patients included patients over the age of 18 and patients with a formal diagnosis of NF2-SWN (with either uni- or bilateral vestibular schwannomas). Exclusion criteria included patients under the age of 18, patients with an undocumented history of NF2-SWN, scans without contrast, scans with large voxel sizes or scan sequences that did not allow for visualization of the inner auditory canal. Inclusion criteria did not include treatment status or presence of other tumors such as meningiomas; therefore, the patient data represented both pre- and posttreatment scans as well as those with meningiomas.

### 2.2 Images

All imaging was conducted at Yale New Haven Hospital (New Haven, CT) using a combination of 1.5T Toshiba (one scan), 3T Siemens (seven scans), or GE systems (two scans). Volumetric analysis was performed on high-resolution, thin-slice axial T1 post-contrast MRI scans. The voxel sizes of each scan are detailed in Table 1.

**Table 1:** Voxel sizes of the original scans used.

Voxel size (mm)	Number of scans
0.5 × 0.5 × 0.75	1
0.4688 × 0.4688 × 0.9	2
0.3438 × 0.3438 × 1.0	1
0.5078 × 0.5078 × 0.9	2
0.4688 × 0.4688 × 1.2	1
0.4297 × 0.4297 × 0.9	1
0.375 × 0.375 × 1.0	1
0.4683 × 0.4683 × 1.0	1

### 2.3 Manual segmentation

Three researchers (M.S., N.J., and S.L.) independently segmented the 17 original tumors using Simpleware (Synopsys, Mountain View, CA) “Paint by Threshold” tool. Each tumor mask was reviewed and corrected by a neuroradiologist (M.H.J.). The final volume of each segmented tumor was then measured using the software's volume measurement tool to establish a baseline prior to resampling.

### 2.4 3D model creation

In addition to manual segmentation, each tumor was segmented using the AI-based auto-segmentation tool in Simpleware, as previously described and validated by Jester et al. Our segmentation process utilized the Simpleware platform by Synopsys, which integrates proprietary AI-powered auto-segmentation tools. These tools leverage a convolutional neural network trained on large, domain-specific datasets to accurately and efficiently segment complex anatomical structures, including VS tumors. To ensure reliability, we validated Simpleware's output against ground truth annotations using metrics such as the Dice coefficient (7). The volume of each AI-generated tumor mask was calculated using the same volume measurement tool.

### 2.5 Resampling

To evaluate the effect of voxel resolution on volumetric accuracy, the resampling function within the image processing software was used to lower the resolution of the original MRI images by resampling them to larger voxel sizes via linear interpolation between neighboring voxels. Following voxel size modification, the researchers re-segmented the tumors using both manual and AI-based methods as described above, and the resulting tumor volumes were measured.

We resampled each scan to four different voxel sizes (0.5 × 0.5 × 0.8 mm, 0.8 × 0.8 × 0.9 mm, 0.8 × 0.8 × 1.6 mm, and 1.2 × 0.9 × 4.0 mm) based on their common use in clinical 3T internal auditory canal MRI protocols (9). This range allows us to evaluate the trade-off between spatial resolution and segmentation accuracy in tumor volume estimation.

Segmentation accuracy across different voxel sizes and methods (AI vs. manual) was evaluated by calculating the percentage change in volume, Dice similarity coefficient scores, and Hausdorff distances, using the original tumor mask as the reference.

### 2.6 Data analysis

Statistical significance was assessed using the Wilcoxon signed-rank test, comparing resampled tumor volumes across different voxel sizes. Additionally, we applied this test to evaluate differences between AI-based and manual segmentation methods in terms of percentage volume change, Dice similarity coefficients, and Hausdorff distances. All statistical analyses were performed using R (R Core Team), and visualization of volume differences, Dice similarity coefficients, and Hausdorff distances was conducted using ggplot2 and tidyverse (10, 11) for comparative analysis.

## 3 Results

### 3.1 Effect of voxel size on manual segmentation volume

**Figure 1:**
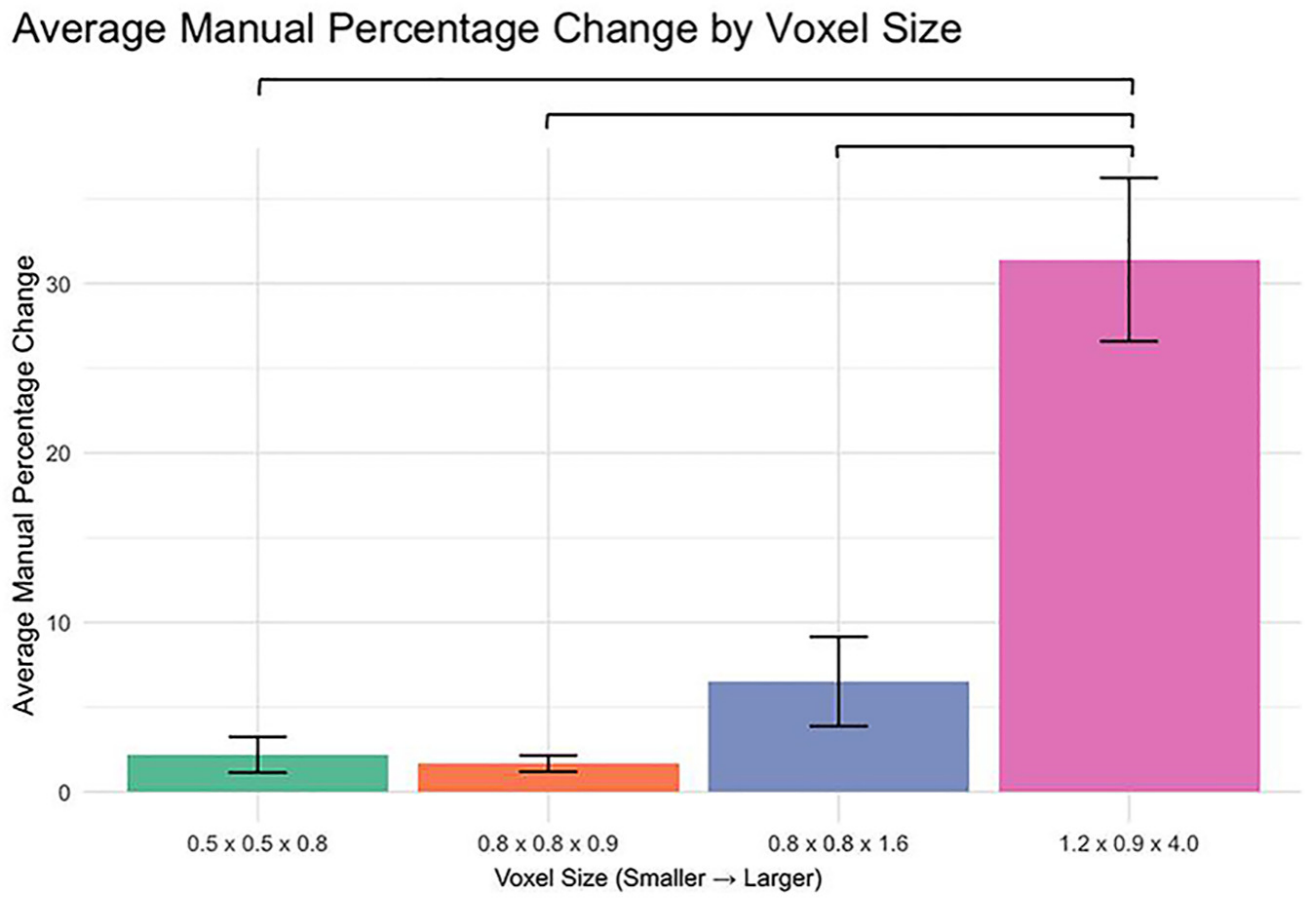
Percent change in manual segmentation volume by voxel size.

The analysis of percent change in manual segmentation volume across different voxel sizes revealed significant differences when comparing the 1.2 × 0.9 × 4.0 mm voxel size to each of the smaller voxel sizes (Figure 1). Specifically:

0.5 × 0.5 × 0.8 mm vs. 1.2 × 0.9 × 4.0 mm: *p* < 0.0001
0.8 × 0.8 × 0.9 mm vs. 1.2 × 0.9 × 4.0 mm: *p* < 0.0001
0.8 × 0.8 × 1.6 mm vs. 1.2 × 0.9 × 4.0 mm: *p* < 0.0001

No significant differences were observed between the smaller voxel sizes (*p* > 0.05).

### 3.2 Comparison of AI and manual segmentation volume changes

**Figure 2:**
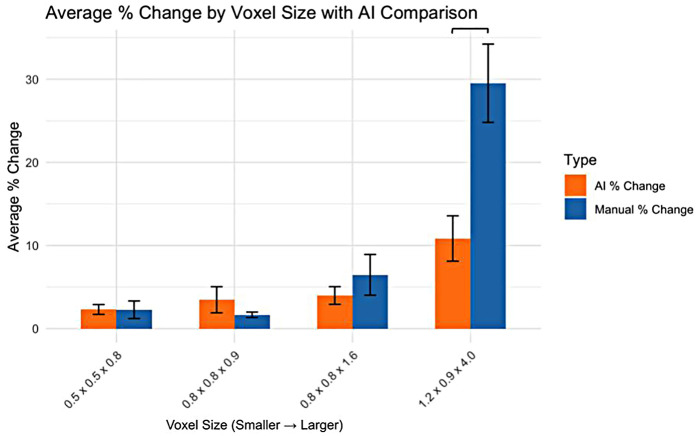
AI vs. Manual Segmentation: Percent Change in Volume by Voxel Size.

For the smaller voxel sizes, no significant differences were observed in the percentage change in volume between AI and manual segmentation methods (Figure 2). However, for the 1.2 × 0.9 × 4.0 mm voxel size, a significant difference was observed, with AI segmentation showing a lower percentage change in volume compared to manual segmentation.

### 3.3 Manual segmentation accuracy (dice score analysis)

**Figure 3:**
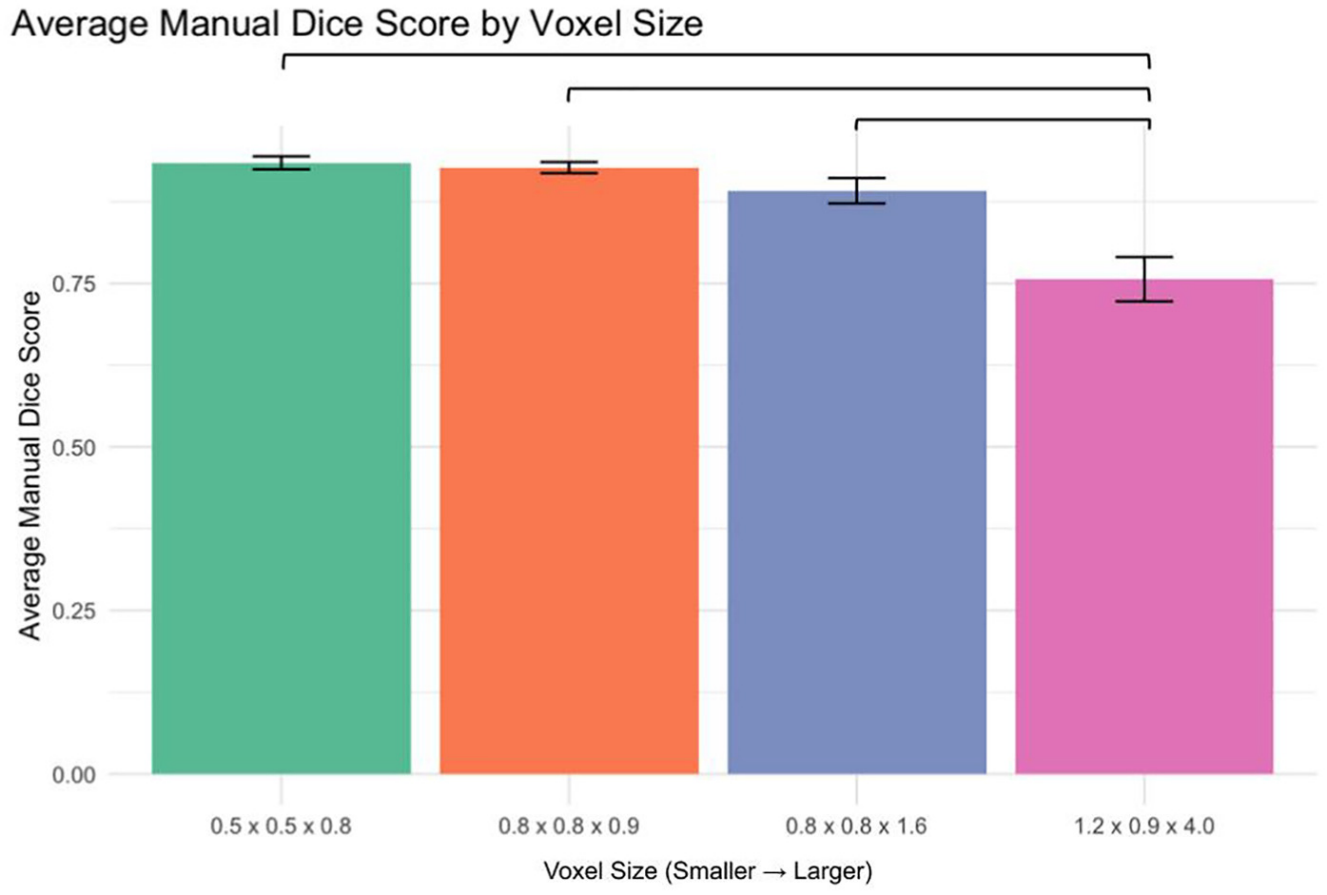
Manual segmentation dice score by voxel size.

Analysis of manual segmentation Dice scores demonstrated significant differences when comparing the 1.2 × 0.9 × 4.0 mm voxel size to smaller voxel sizes (Figure 3):

0.5 × 0.5 × 0.8 mm vs. 1.2 × 0.9 × 4.0 mm: *p* < 0.0001
0.8 × 0.8 × 0.9 mm vs. 1.2 × 0.9 × 4.0 mm: *p* < 0.0001
0.8 × 0.8 × 1.6 mm vs. 1.2 × 0.9 × 4.0 mm: *p* < 0.0001

No significant differences were found between smaller voxel sizes (*p* > 0.05).

### 3.4 Comparison of AI and manual dice scores

**Figure 4:**
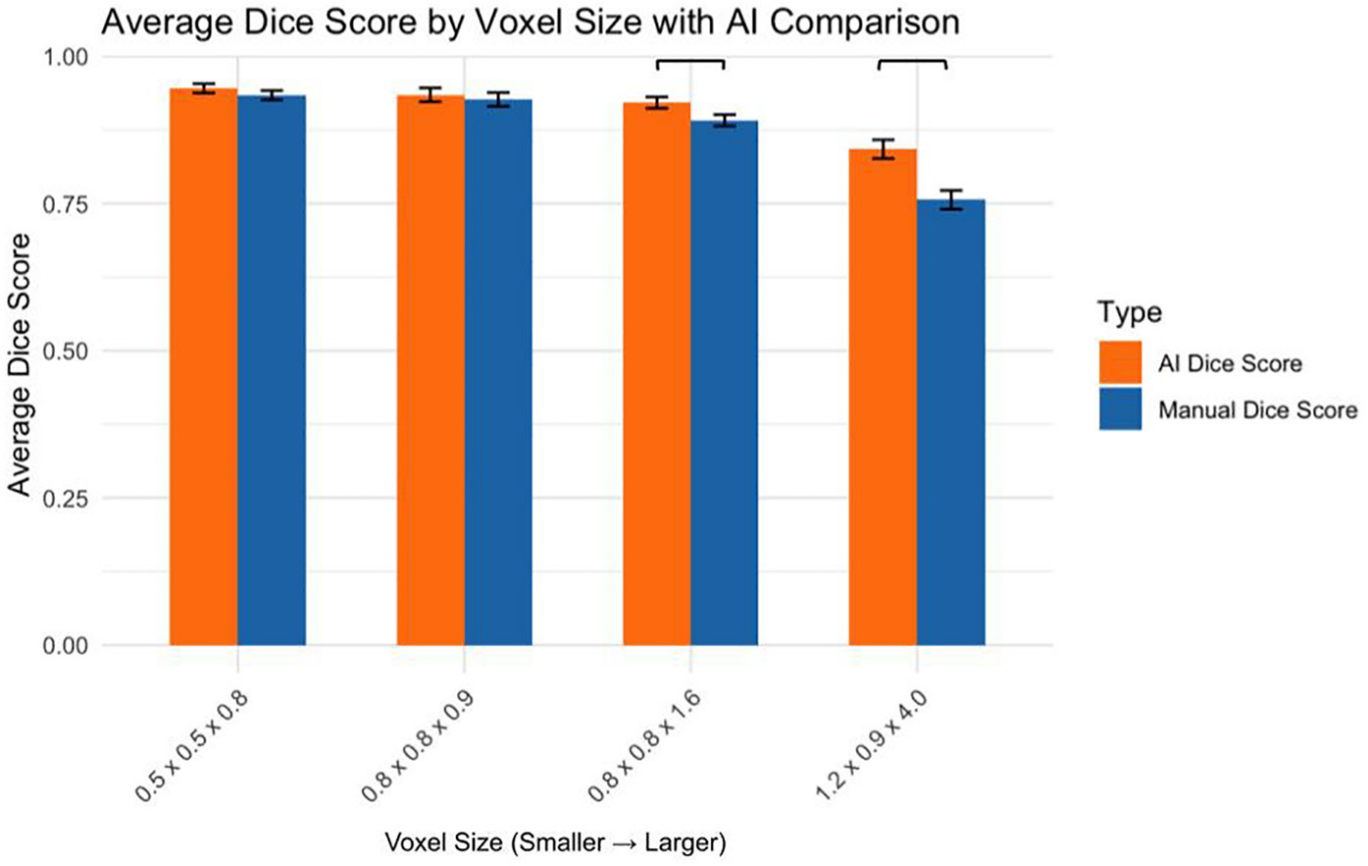
AI vs. Manual Segmentation: Dice Score by Voxel Size.

The AI and manual segmentation Dice scores were compared across voxel sizes (Figure 4):

0.5 × 0.5 × 0.8 mm: AI significantly outperformed manual (*p* < 0.05)
0.8 × 0.8 × 0.9 mm: No significant difference (*p* > 0.05)
0.8 × 0.8 × 1.6 mm: AI significantly outperformed manual (*p* < 0.01)
1.2 × 0.9 × 4.0 mm: AI significantly outperformed manual (*p* < 0.05)

### 3.5 Manual segmentation accuracy (Hausdorff distance analysis)

**Figure 5:**
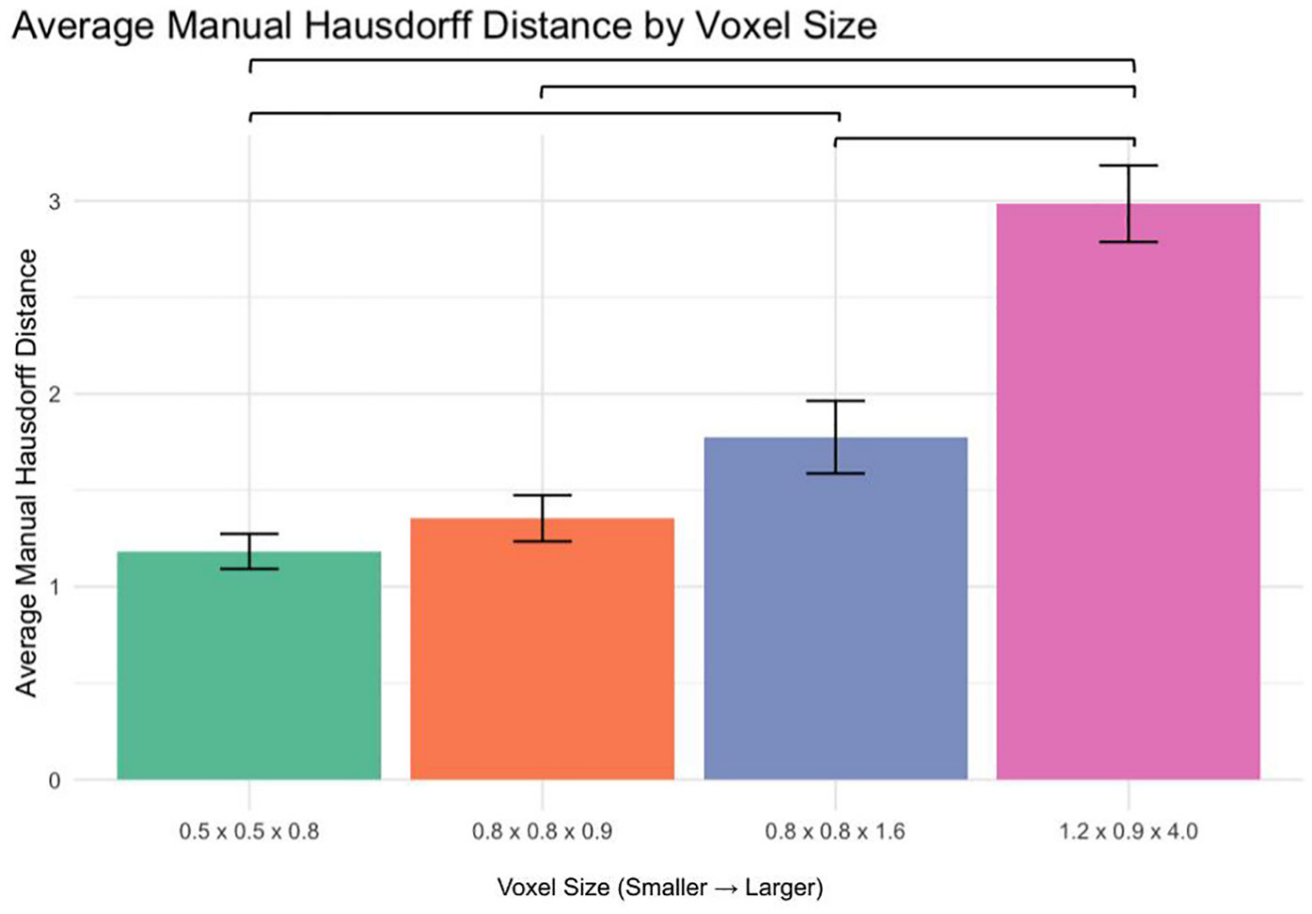
Manual segmentation hausdorff distance by voxel size.

The average Hausdorff distance was analyzed to assess segmentation accuracy (Figure 5):

0.5 × 0.5 × 0.8 mm vs. 1.2 × 0.9 × 4.0 mm: *p* < 0.0001
0.8 × 0.8 × 0.9 mm vs. 1.2 × 0.9 × 4.0 mm: *p* < 0.0001
0.8 × 0.8 × 1.6 mm vs. 1.2 × 0.9 × 4.0 mm: *p* < 0.0001

No significant differences were observed between 0.5 × 0.5 × 0.8 mm and 0.8 × 0.8 × 0.9 mm or 0.8 × 0.8 × 0.9 mm and 0.8 × 0.8 × 1.6 mm (*p* > 0.05). However, a significant difference was found between 0.5 × 0.5 × 0.8 mm and 0.8 × 0.8 × 1.6 mm (*p* < 0.01).

### 3.6 Comparison of AI and manual hausdorff distances

**Figure 6:**
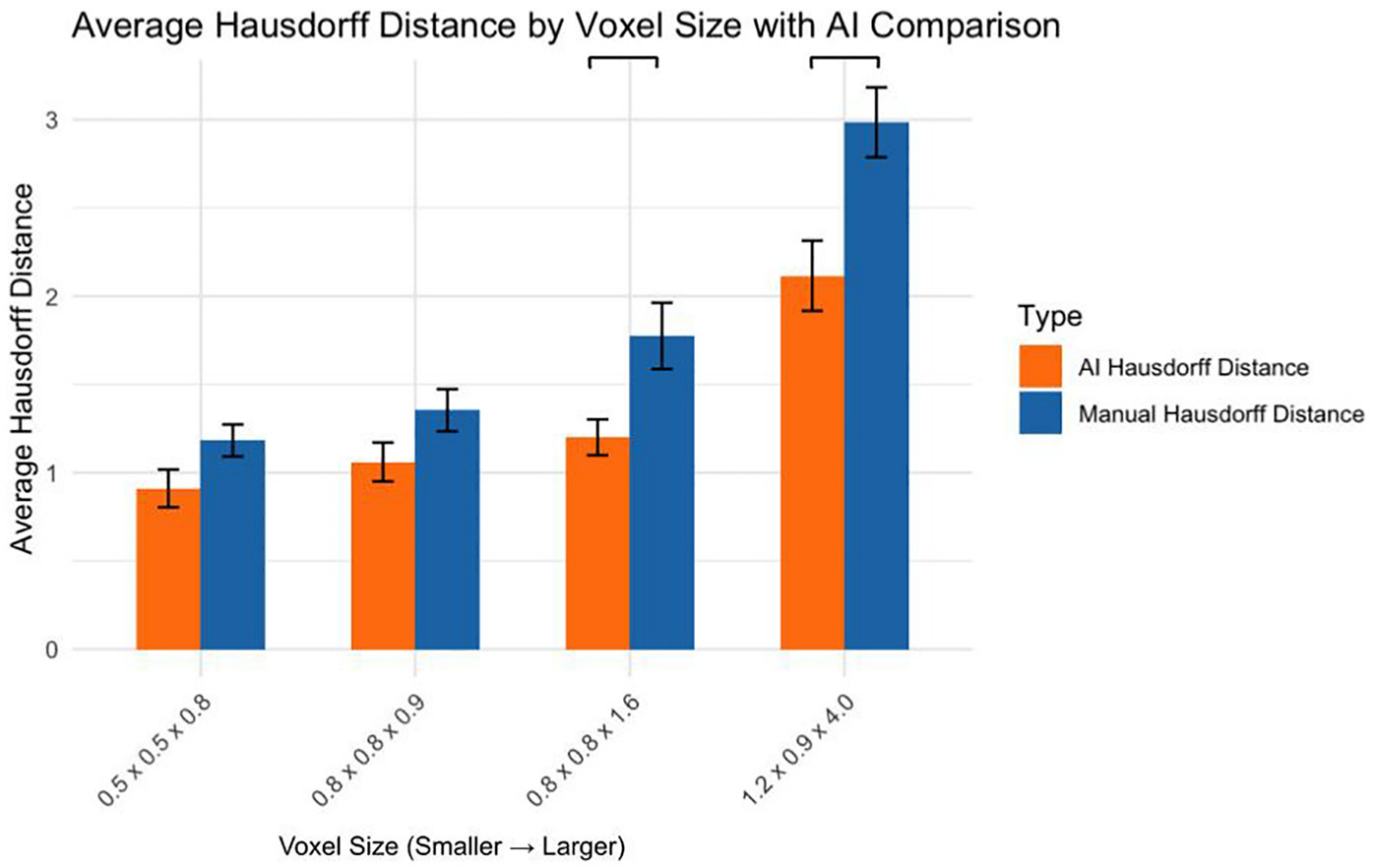
AI vs. Manual Segmentation: Hausdorff Distance by Voxel Size.

Comparing AI and manual segmentation methods revealed (Figure 6):

0.5 × 0.5 × 0.8 mm: No significant difference (*p* > 0.05)
0.8 × 0.8 × 0.9 mm: No significant difference (*p* > 0.05)
0.8 × 0.8 × 1.6 mm: AI significantly outperformed manual (*p* < 0.05)
1.2 × 0.9 × 4.0 mm: AI significantly outperformed manual (*p* < 0.001)

## 4 Discussion

This study systematically evaluated how MRI voxel size affects the accuracy of manual and AI-driven volumetric segmentation of VS in patients with NF2-SWN, a genetically driven disorder characterized by bilateral vestibular tumors. Across the dataset, we observed a clear inverse relationship between voxel size and segmentation accuracy. Larger voxels (1.2 × 0.9 × 4.0 mm) were associated with significant degradation in Dice similarity scores and increased Hausdorff distances, especially in manual segmentations. Smaller voxel sizes (0.5 × 0.5 × 0.8 mm, 0.8 × 0.8 × 0.9 mm) consistently produced more accurate tumor delineations.

The impact of voxel resolution on segmentation accuracy has been well-documented across various imaging modalities. In MRI, voxel size directly influences spatial resolution, with smaller voxels enabling finer anatomical detail capture (12). This is particularly crucial for delineating small or irregularly shaped tumors, where boundary precision is essential. Studies have shown that reduced voxel sizes can enhance the accuracy of tumor segmentation by minimizing partial volume effects and improving edge definition [(13, 14); Dan et al., 2019; (15)]. However, high-resolution imaging comes with trade-offs, including increased data volume and processing time (16). These longer scan times can be particularly challenging in clinical settings involving pediatric, elderly, or otherwise non-compliant patient populations, where motion artifacts and discomfort may compromise image quality. The benefits of higher resolution may plateau beyond a certain point, especially when the tumor size is small or the contrast between tumor and surrounding tissue is low. In such cases, the improvement in segmentation accuracy may not justify the additional computational burden. Therefore, optimizing voxel resolution requires balancing the need for detail with the practical constraints of imaging and processing capabilities.

**Figure 7:**
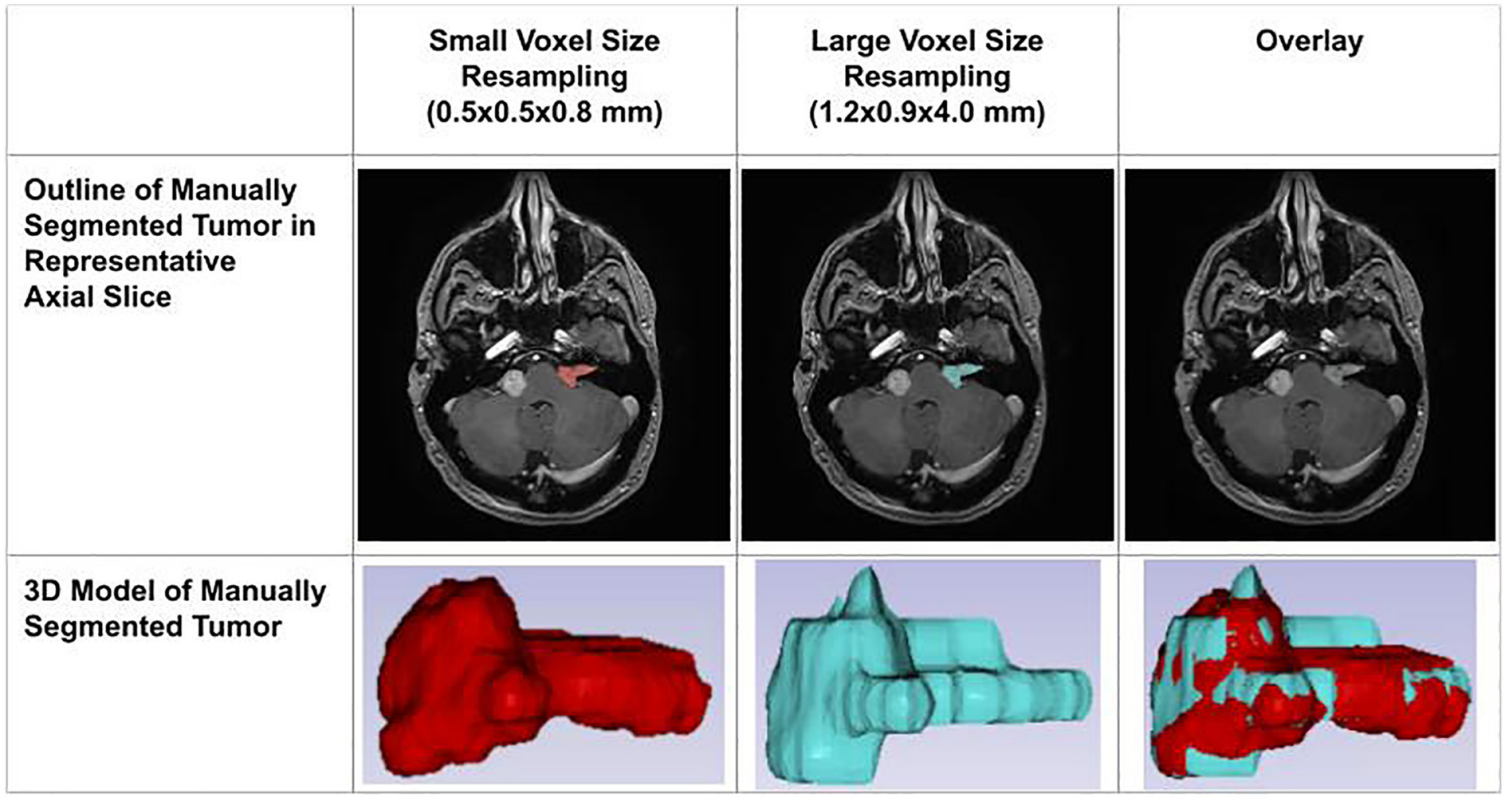
Impact of voxel resolution on tumor segmentation. ***Left***: Small-voxel size (0.5 × 0.5 × 0.8 mm) axial slice and 3D tumor model. ***Center***: Large-voxel size (1.2 × 0.9 × 4.0 mm) axial slice and model. ***Right***: Overdays reveal discrepancies between high- and low-resolution segmentations in both 2D and 3D.

**Figure 8:**
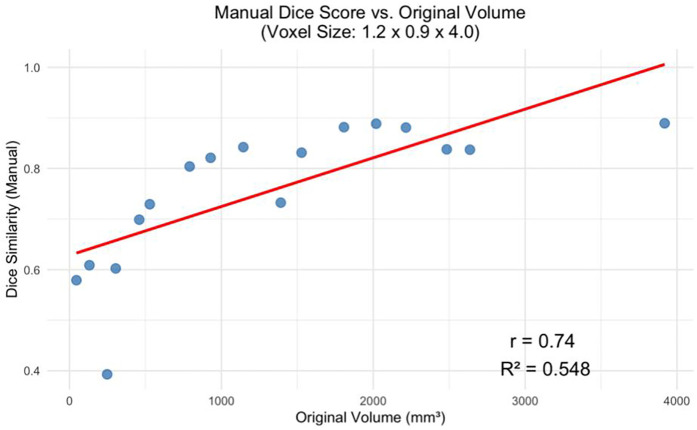
Manual dice score for the greatest voxel size (1.2 × 0.9 × 4.0 mm) vs. Tumor Volume at Original Resolution (*p* < 0.001).

**Figure 9:**
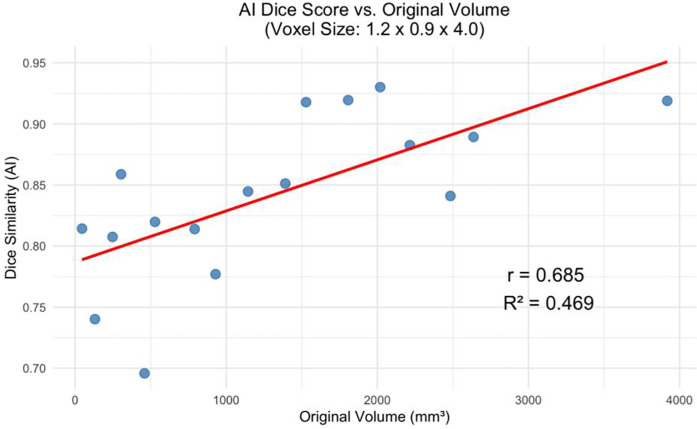
AI dice score for the greatest voxel size (1.2 × 0.9 × 4.0 mm) vs. Tumor Volume at Original Resolution (*p* < 0.01).

The decline in segmentation performance at lower resolutions was more pronounced in manual contours (Figure 7), whereas AI-driven methods demonstrated greater robustness. Notably, AI segmentations showed reduced variability and maintained relatively high accuracy even at larger voxel sizes. Dice similarity scores for AI segmentations also exhibited a significant positive correlation with tumor volume (Figure 8), consistent with the observed trend in manual segmentation (Figure 9). This volume-dependent accuracy underscores that larger tumors are easier to segment, likely due to their lower surface-area-to-volume ratio, which reduces the relative influence of partial volume effects and boundary ambiguity. This volume-dependent accuracy likely arises because larger tumors have a lower surface-area-to-volume ratio, which reduces the relative influence of boundary ambiguities and voxel-level noise.

While both segmentation approaches performed well on large tumors, smaller lesions posed a greater challenge. For the smallest tumors in our dataset, Dice scores often fell below 0.80 for manual segmentation and hovered around 0.75–0.85 for AI. This reduced performance may be partly attributable to the fact that, in smaller tumors, boundary voxels constitute a larger proportion of the total volume. As a result, even minor inaccuracies along the tumor edge disproportionately impact Dice similarity scores. These findings align with prior observations that small tumor volumes are more vulnerable to inter-observer variability and voxel-level segmentation noise, particularly when spatial resolution is limited (26).

Importantly, these results also highlight the limitations of Dice similarity as a standalone metric. Because Dice is inherently volume-weighted, its value is strongly influenced by tumor size. A modest absolute error may have minimal impact on Dice in large tumors but can dramatically reduce it in smaller lesions where boundary voxels dominate. Therefore, we also employed Hausdorff distance as a complementary measure of segmentation accuracy. Unlike Dice, the Hausdorff distance captures the maximum boundary discrepancy between the predicted and ground truth contours, offering a more precise assessment of spatial alignment—especially at tumor margins. This metric is particularly relevant in clinical contexts, such as surgical and radiotherapy planning, where accurate boundary delineation is more critical than overall volume overlap. Recent studies have advocated for hybrid evaluation metrics that combine region-based and boundary-based assessments to more comprehensively reflect segmentation performance (17).

## 5 Limitations

Despite the performance advantages of AI segmentation, several limitations should be acknowledged. First, manual delineation—used here as the reference standard—carries inherent subjectivity. Even among experts, tumor boundaries are variably defined, particularly in low-contrast regions or for irregular tumor shapes. Studies have reported inter-rater differences of up to 20%–30% in VS volumes, highlighting the imprecision of manual ground truth (18, 19). Additionally, the ground truth itself may be affected by the partial volume effect, wherein a single voxel contains a mixture of tissues, leading to boundary ambiguity (20, 25).

AI models are not immune to these challenges. While they mitigate inter-rater variability, they may inherit biases from their training labels and are sensitive to input quality. Many deep learning models—including those tested in similar contexts—are trained on single-institution datasets, raising concerns about generalizability across scanner types, sequences, and patient populations (21). Furthermore, conventional segmentation networks, like U-Net, often misclassify boundary voxels due to class imbalance, smoothing artifacts, and the inherently fuzzy nature of MRI tumor margins (17, 22). This is especially problematic when tumors lie adjacent to critical structures such as the cochlea or brainstem, where small boundary errors may impact treatment decisions.

Another limitation is the heterogeneity of our dataset in terms of scanner type, manufacturer, and acquisition protocol. Although this diversity could, in theory, enhance the generalizability of AI segmentation tools, our small sample size precludes drawing robust conclusions about cross-scanner performance. The lack of harmonization protocols—such as intensity normalization or bias field correction—may have introduced confounding variability that affected both manual and AI-based segmentation outcomes. However, since manual clinical segmenters routinely encounter these same challenges in real-world settings, the results remain clinically relevant.

Although the magnet strength (1.5T, 3T) and sequence type (axial T1 post-contrast) were consistent across scans, echo time (TE), repetition time (TR), and other acquisition parameters were not standardized due to differences in scanner vendors and institutional protocols. Such variability may influence image contrast and signal-to-noise ratios, potentially affecting segmentation accuracy and generalizability.

While proprietary software was used for manual delineation, each voxel was individually painted to define tumor boundaries—a functionality present in widely available open-source platforms as well. This approach supports reproducibility, as the segmentation method can be replicated using common voxel-wise editing tools.

## 6 Clinical implications

Accurate, reproducible volumetric segmentation is essential in NF2-SWN management, where treatment is often guided by serial MRI tracking. Manual segmentation—while familiar—can be time-consuming and inconsistent, particularly in longitudinal assessments. AI-based tools offer a solution to this bottleneck, enabling rapid and standardized tumor volume estimation. This is particularly valuable in the context of the “wait-and-scan” strategy, where detecting subtle growth trends is critical. In clinical practice, a 20% increase in tumor volume is typically used to define progression, though this threshold is partly dictated by measurement reproducibility (18). AI tools that reduce segmentation variability could allow earlier detection of significant changes, especially in borderline cases, and may help reduce unnecessary interventions by distinguishing true progression from segmentation noise.

Moreover, high-precision segmentation is crucial for planning radiosurgery or microsurgical resection. Misestimating tumor boundaries can lead to undertreatment or overtreatment, particularly when critical neurovascular structures are involved. Incorporating boundary-aware AI models into pre-operative planning may improve outcomes by ensuring adequate treatment margins while minimizing collateral damage.

## 7 Future directions

Several deep learning innovations are emerging to address the limitations of conventional segmentation approaches. Boundary-aware loss functions—such as Boundary Loss (23), Distance-based Loss (17), and Adaptive Edge-Enhanced Dice (24)—have shown promise in improving contour accuracy by penalizing boundary errors more directly than region-based losses. These functions focus learning on thin structures and edge fidelity, especially beneficial for small or complex tumors.

Complementary architectural solutions are also gaining traction. Multi-scale CNNs and patch-wise segmentation models, which process high-resolution crops alongside full-field views, have improved performance in segmenting tumors with irregular margins or infiltrative patterns (Sweetline et al., 2024). Incorporating these designs into AI tools for NF2-related tumor segmentation may further enhance boundary precision and support clinical decision-making.

To improve generalizability and robustness, future studies must expand sample sizes and ensure diversity across institutions, MRI vendors, and imaging protocols. Our dataset included images from multiple scanner types (1.5T Toshiba, 3T Siemens, and GE systems), but the study was not powered to assess their individual effects. A larger cohort would enable formal subgroup analyses to quantify how magnetic field strength, vendor-specific image reconstruction, and pulse sequence parameters impact segmentation accuracy. Such insights could inform scanner-specific calibration strategies or lead to the development of harmonization pipelines to normalize image characteristics before segmentation.

Standardization and external validation will also be critical as models move from development into clinical deployment. Most current studies lack rigorous multi-center testing, and performance may degrade on out-of-distribution inputs. Future efforts should prioritize the development of robust, scanner-agnostic models trained on heterogeneous datasets that reflect real-world variability in anatomy, imaging quality, and post-surgical alterations. Uncertainty quantification—such as confidence maps—could further support clinician oversight in ambiguous cases, fostering a human-in-the-loop paradigm that balances automation with expert review.

Ultimately, integrating AI segmentation into clinical workflows could transform care for NF2 patients. Automated delineations immediately following MRI acquisition, verified and adjusted by radiologists, would streamline reporting and improve accuracy. Such tools also open new research avenues, including radiomic analysis and predictive modeling of tumor behavior.

## Data Availability

The datasets presented in this article are not readily available because the imaging and segmentations are not available to anyone as they consist of MRIs of the head which include faces. Requests to access the datasets should be directed to rithvik.ghankot@yale.edu.
